# Minimum accepted competency examination: test item analysis

**DOI:** 10.1186/s12909-022-03475-8

**Published:** 2022-05-25

**Authors:** Paddy McCrossan, Alf Nicholson, Naomi McCallion

**Affiliations:** 1 Academic Clinical Lecturer, Queen’s University Belfast, 97 Lisburn Rd, Belfast, BT9 7BL UK; 2 Head of School of Medicine, Royal College of Surgeons in Ireland Bahrain, Building No. 2441, Rd 2835, Busaiteen 228, Manama, 15503 Bahrain; 3 Associate Professor Department of Paediatrics, Royal College of Surgeons in Ireland, 123 St Stephen’s Green, Dublin 2, Ireland

**Keywords:** Medical education, Assessment, Standard setting, Multiple choice questions, Competence

## Abstract

**Background:**

To ascertain if undergraduate medical students attain adequate knowledge to practice in paediatrics, we designed the minimum accepted competency (MAC) examination. This was a set of MCQ’s designed to test the most basic, ‘must know’ knowledge as determined by non-faculty paediatric clinicians. Only two-thirds of undergraduate students passed this exam, despite 96% of the same cohort passing their official university paediatric examination.

We aim to describe the psychometric properties of the MAC examination to explore why there was a difference in student performance between these two assessments which should, in theory, be testing the same subject area. We will also investigate if the MAC examination is a potentially reliable method of assessing undergraduate knowledge.

**Methods:**

The MAC examination was sat by three groups of undergraduate medical students and paediatric trainee doctors. Test item analysis was performed using facility index, discrimination index and Cronbach’s alpha.

**Results:**

Test item difficulty on the MAC between each group was positively correlated. Correlation of item difficulty with the standard set for each item showed a statistically significant positive relationship. However, for 10 of the items, the mean score achieved by the candidates did not even reach two standard deviations below the standard set by the faculty. Medical students outperformed the trainee doctors on three items. 18 of 30 items achieved a discrimination index > 0.2. Cronbach’s alpha ranged from 0.22–0.59.

**Conclusion:**

Despite faculty correctly judging that this would be a difficult paper for the candidates, there were a significant number of items on which students performed particularly badly. It is possible that the clinical emphasis in these non-faculty derived questions was juxtaposed with the factual recall often required for university examinations.

The MAC examination highlights the difference in the level of knowledge expected of a junior doctor starting work in paediatrics between faculty and non-faculty clinicians and can identify gaps between the current curriculum and the ‘hidden curriculum’ required for real world clinical practice. The faculty comprises physicians in employment by the University whose role it is to design the paediatric curriculum and deliver teaching to undergraduate students. Non-faculty clinicians are paediatric physicians who work soley as clinicians with no affiliation to an educational institution.

The concept of a MAC examination to test basic medical knowledge is feasible and the study presented is an encouraging first step towards this method of assessment.

**Supplementary information:**

The online version contains supplementary material available at 10.1186/s12909-022-03475-8.

## Background

This study investigates whether undergraduate training adequately prepares doctors with the required knowledge to practice paediatrics at the early senior house officer (SHO) level. SHO is the level at which a doctor will have their first experience in paediatrics, usually 12–24 months post-graduation from medical school. Prior to commencing clinical, post-graduate work, all a doctor’s knowledge on the subject is based upon their undergraduate experience and teaching. The hypothesis is that there are areas of knowledge and skills that are important at SHO level that are either missed, not highlighted or not incentivised in the undergraduate curriculum. This may result in students successfully passing undergraduate assessment and then entering the workplace with crucial knowledge deficits [1].

To that end, we designed a novel approach to assessment in which question content was provided by non-faculty paediatric clinicians with the remit of ‘must know basic knowledge for a doctor starting work in paediatrics,’ (i.e., SHO). This examination was named, Minimum Accepted Competency (MAC) and the methodology for creating the test and gross examination results have previously been described [2]. The logic behind this approach is that we wanted to capture a finite amount of the most relevant crucial knowledge that students should be assessed in, thereby equipping them with the knowledge to tackle most clinical tasks early in in their career and provide a solid platform on which to build. As the questions targeted essential competencies for practice, most candidates should be able to answer them correctly. The passing score for the examination was therefore expected to be appropriately high (i.e., candidate must get most of the questions correct to pass the examination). This would ensure that no student graduated medical school and potentially start work in paediatrics without a firm grasp of this essentiallevel of knowledge.

However, the passing score for the MAC examination was calculated at 41.2% (much lower than anticipated considering the genesis of this assessment). The average score achieved by undergraduate students was 46% with only two thirds reaching the pass mark, whereas in the university paediatric examination, 96% of students passed. The average score achieved by postgraduate SHO’s was 64% [2]. Therefore, we concluded that there must be a gap in the expectation of knowledge between non-faculty paediatricians and the academic paediatric faculty. Overall candidates did not have the required knowledge and so performed poorly in this type of assessment.

We will now delve deeper into the content of the MAC examination and describe the psychometric results (as opposed to simply the gross score obtained by each candidate) in a bid to explain why there was such a difference in student performance between these two assessments which should, in theory, be testing the same subject area. We will identify those test items which were particularly difficult for the candidates and provide potential reasons for this including cross checking the current undergraduate curriculum.

## Methodology

### Designing the MAC examination

The methodology for designing the content, standard setting and administering the MAC examination has already been described [2]. In brief, paediatric non-faculty clinicians (who do not have a role in setting examinations) were asked to generate questions based on “must know” information that, in their opinion, was necessary for every junior doctor starting their first post in paediatrics. The questions were reformatted to a ‘single best answer’ MCQ structure to match the question format in use at the time for paediatric undergraduates at Royal College of Surgeons in Ireland (RCSI). A bank of questions was created, and a random number generator was used to choose 30 questions to form the research examination (MAC) paper. The sequence of questions is the same for each group that set the MAC exam. The questions were standard-set by the undergraduate academic paediatric faculty of the RCSI at a standard-setting meeting for the university’s paediatric written examination. Academic staff participating in the standard-setting were blinded as to whether questions formed part of the official university written examination or comprised part of this research study. Using a modified Angoff technique, 9 members of the RCSI faculty calculated a passing score of 41.2%, equating to a passing score of 13/30 on the MAC examination [2].

### Participants

Undergraduate students were recruited from two universities: RCSI (Dublin) and Queen’s University Belfast (QUB) from June 2016-June 2018. RCSI students from the penultimate year of university (during which they complete their paediatric teaching) were invited to attend for a mock examination (the ‘MAC’ exam), one week before sitting their university written examination at the end of the academic year [Year 1 RCSI]. The following year, RCSI students were invited to sit the MAC examination at the end of their 6-week paediatric clinical attachment [Year 2 RCSI]. QUB students from the penultimate year of the medical course (the year in which they complete their paediatric teaching) were invited to sit the MAC examination at the end of their paediatric clinical attachment [QUB]. Both universities offer an equivalent paediatric experience, both lasting 6 weeks with a mixture of didactic lectures, tutorials, and secondary care (hospital based) placements. All SHOs currently enrolled in the Irish Basic Specialist Training (BST) [3] scheme for paediatrics, were approached to sit the MAC examination during the first paediatric training day of the new academic year at which point they had been working in paediatrics for 3 months [BST]. See Table 1 for total numbers of participants.

**Table 1:** Number of participants in the MAC examination from each group

Participant group	Year 1 RCSI	Year 2 RCSI	QUB	BST
Number of participants	198	168	54	58

### Test item analysis

Once the results were collected, we performed test item analysis, facility index [F], discrimination index [D] and Cronbach’s Alpha. Test item analysis is used to identify which questions were most difficult and whether this is consistent between the studied groups.

Item facility (F) is measured by the proportion of candidates who correctly answered a given test item. Therefore, a test item, which was difficult, is flagged by a low percentage of candidates giving a correct response to that item [4]. Some authors have suggested that a test item should attempt to have a facility index (F) in the range between 0.3–0.7, as questions which are too easy or too difficult may be unable to differentiate between candidates [5]. We used this measurement to ascertain which test items the candidates found most difficult. We used Spearman’s rank to correlate item difficulty between the different groups who sat the examination to determine if the same questions were found difficult between the groups (one assessment of a tests reliability). We also correlated item difficulty with that same item’s standard set using Pearson correlation coefficient.

The discrimination index (D) is the point-biserial correlation coefficient between the test item and the mean score. This examines whether a test item is able to distinguish between a student of high ability and those with low ability [6, 7]. A D < 0 (i.e., a negative value) would mean that the best students got this question wrong more often than the worst students. D = 0 would mean it was a poor discriminator between good and bad students. A test item, which achieves D > 0.2 is deemed ‘good’ and therefore a re-useable question for future sittings of that examination [5]. We included this measurement as a tool to appraise the suitability of the test items for potential future use.

The Cronbach’s alpha is a measure of internal consistency. It is a description of the extent to which all the items in a test measure the same concept and is, therefore, a way of expressing the inter-relatedness of the items within the test [8]. Cronbach’s alpha is a tool commonly used to assess the reproducibility of an assessment [8] and so it was included in our analysis as another means to determine the reliability of the MAC as an examination.

In the absence of any further guideline, we used the five most difficult questions from the MAC examination as examples to consider if there is a pattern between the studied groups' performance. We also used the arbitrary measure of two standard deviations below the faculty-derived standard set for each question as a marker of particularly poor performance if not met.

We analysed in more detail the items which we have identified as particularly difficult for the candidates and try to explain why this might have been the case. We indicate whether the answer to the question could have been found in the university paediatric curriculum (and therefore arguably should have been prepared for by the undergraduate students who sat the MAC exam).

Institutional ethical approval did not allow for a direct comparison of the individual results of QUB and RCSI students.

## Results

**Fig. 1:**
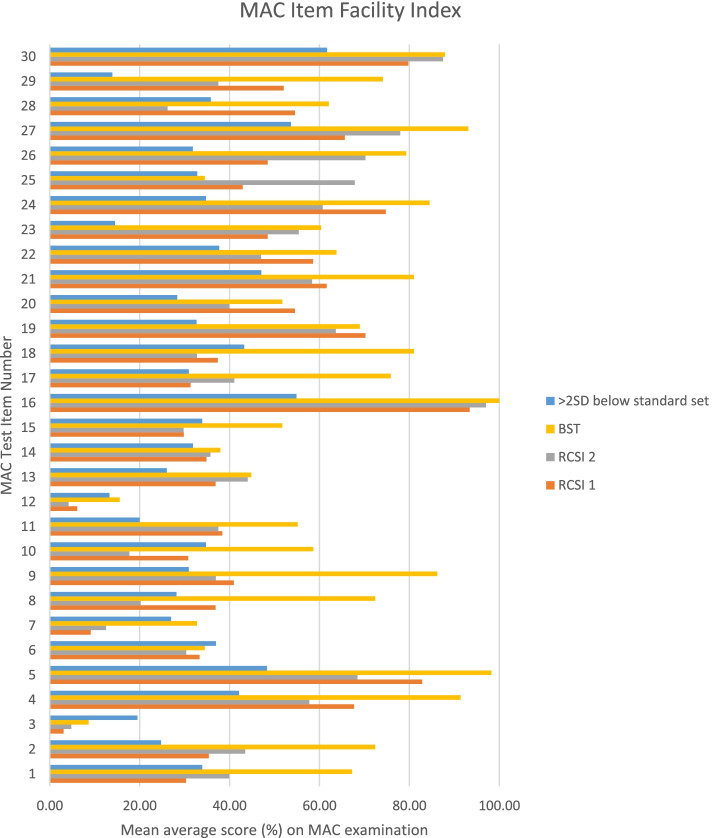
Bar chart illustrating the mean average score achieved on each test item (i.e. facility index [F]) of the MAC examination by each study group alongside a measure of 2 standard deviations (SD) below the RCSI faculty derived standard set score for that test item

A full description of the discrimination index and facility index for each test item is found in appendix 1. When the test item difficulty is ranked within each group, a Spearman’s rank correlation indicates a positive correlation between Year 1 RCSI and Year 2 RCSI 0.80 (*p* < 0.01), Year 1 RCSI and BST 0.73 (*P* < 0.01)) and Year 2 and BST 0.66 (P < 0.01) (see Fig. 1).

Correlations of item difficulty with standard set mark for each item on the MAC exam show a statistically significant positive relationship for both undergraduate groups. For Year 1 RCSI, the Pearson correlation coefficient was 0.66 (*p* < 0.01), for Year 2 RCSI, it was 0.62 (*p* < 0.01). It was not appropriate to compare the BST cohort with the standard set mark as this was standard set for undergraduates. Therefore, overall, the most difficult questions as set by the faculty proved to be most difficult for the examinees also. However, when we look in more detail, for items 1,3,6,7,8,10,12, 15,18 and 28, the mean score for one or more of the study groups did not even reach 2 standard deviations below the standard set by the faculty. Therefore, by this measure, performance on these questions fell considerably below the standard expected. Further examination of these questions is merited.

The Cronbach alpha for Year 1 RCSI, Year 2 RCSI, QUB and the SHOs was 0.36, 0.29, 0.22 and 0.59 respectively.

From the results illustrated in Table 2, question item's 3,12 and 7 were the three questions universally difficult across all three groups.

**Table 2:** Top 5 most difficult questions

Difficulty rank of question	1	2	3	4	5
Year 1 RCSI MAC test item	12	3	7	15	1
Year 2 RCSI MAC test item	12	3	7	28	15

We therefore identified test items 3,12 and 7 as items worthy of further analysis along with test items 1,6,8,10,15,18 and 28. We investigated these questions further with regards the current curriculum to ascertain why they appeared to cause difficulty (see Table 3). We also investigated test items 19,20, and 25 (see Table 4) in more detail as these were paradoxically answered better by students than SHO’s (even though overall, the SHO’s performed much better than the student groups). A full descriptive analysis of the difficult items is found in appendix 2.

**Table 3:** Analysis of MAC test items identified as ‘particulartly difficult’

Item number	Clinical problem questioned	Mentioned in the curriculum?
1	Focal seizure	No
3	Persistent bacterial bronchitis	No
6	Viral induced wheeze	Yes
7	Obstructive sleep apnoea	No
8	Fluid management	Yes
10	Febrile seizure	Yes
12	Viral respiratory infection	No
15	Gestational age	Yes
18	Bronchiolitis	Yes
28	Congenital heart disease	Yes

**Table 4:** Analysis of MAC test items, which were ‘paradoxically’ answered better by students than SHOs

Item number	Clinical problem questioned	Mentioned in the curriculum?
19	Coarctation of the aorta	Yes
20	New-born screening	Yes
25	Cellulitis	Yes

For both the undergraduate and postgraduate cohorts, most test items performed well with regards discrimination and facility index (see Table 5).

**Table 5:** Proportion of MAC items reaching acceptable thresholds for discrimination index and facility index for each of the participating groups

	**Year 1 RCSI**	**Year 2 RCSI**	**BST**
Number of items with D > 0.2	18	18	22
Number of items with F 0.3-.07	21	19	14

## Discussion

It is unreasonable to expect any undergraduate curriculum to include full coverage of all conditions within paediatrics. Nor would that approach be beneficial to most of the students (only a minority of whom will go on to practice paediatrics). However, it would seem sensible that a student should be expected to acquire the most basic, common, and relevant knowledge first and foremost. The only means by ensuring that they have obtained this knowledge is by assessing it.

The content of the MAC examination was designed by non-academic paediatric clinicians. The examination was given a low passing score when standard set by the university faculty highlighting a disparity between these two groups of consultants with regards knowledge expectations. Despite this low passing score, only two-thirds of students passed the MAC exam, whereas 96% of the same students passed their university paediatric examination [2] Their level of paediatric knowledge reflects the university curriculum, therefore there must be significant gaps between the university curriculum and the ‘hidden’ curriculum as determined by non-academic clinicians.

### Psychometric test item analysis

When the test item difficulty for the MAC examination was ranked within each group, a Spearman’s rank correlation indicated a positive correlation. This illustrates that, broadly, all three groups found the same questions relatively difficult/easy. This adds an element of reliability to the MAC examination as not just are the overall scores equivalent between the two-year groups, but individual question performance is comparable as well.

For a well-prepared group of examinees, item difficulty indices may range from 70–100% [9]. Hence, the passing score is usually higher when the examinee group is more able [10]. When the facility index moves towards high or low from 50%, the discriminating index becomes low. For example, if all the candidates get the question correct or incorrect then there is no way of using that question to discriminate between the best and worst candidates. However, if we omit questions from a potential question bank because the candidates have found it too easy or too hard then, in a sense, the candidates are setting the standard for the papers themselves. We should allow medical professionals to decide what the standard should be (as is the case with our study in using non-faculty clinicians to decide the questions) and then use a rigorous standard setting process such as Angoff to standard set the paper. If it just so happens that the questions are very easy, then this will result in a high passing score and successful candidates must correctly answer a higher-than-normal proportion of questions to pass the exam (as was envisaged for the MAC). Similarly, if the questions happen to be very difficult then this will be corrected for by good standard setting. In that scenario the student need only get a few questions correct to pass.

The Royal College of Paediatrics and Child Health (RCPCH) theory examination faculty find that an MCQ item which scores at least 0.2 on the discrimination index is deemed acceptable and therefore a re-usable question. Questions, which score less than 0.2, are not necessarily discarded, rather they go through a review process at one of the regular board meetings where the theory examiners revise the question to improve its future psychometric value. Interestingly, for the original intention of the MAC examination, one in which we hope the candidate proves to have a basic level of knowledge, this measure would not have been as appropriate or useful. The original intention was to design a paper in which the spread of marks would have been less and more bunched around a relatively high gross score, therein, preventing many of the questions displaying good discriminating ability as most candidates would be getting all of them correct. As it turned out, the paper was found to be relatively difficult with a low overall gross mean score and a wide spread of marks for many of the items which show good ability to discriminate candidates.

It is generally accepted that for an Multiple Choice Question (MCQ) examination, adequate internal consistency is demonstrated by a Cronbach's alpha of at least 0.7 [11]. The low level of Cronbach's alpha seen with the MAC examination may be explained by a poor spread of questions. The examination was limited, for the purpose of improving participation, to only 30 questions and thus it is inevitable that there will be aspects of the curriculum that remain untested. It is known that increasing the number of test items will statistically improve the reliability of MCQ's [12]. High stakes MCQ examinations would generally have many more than 30 items. When compared with the RCSI official written paper, for example, they use 120 items, which would vastly improve the alpha of the MAC.

### Comparison to the curriculum

From the above analysis of the most difficult questions, we can see a pattern emerging. Questions on respiratory paediatrics appear to have been answered poorly. Whilst respiratory conditions are the most common of paediatric presentations, there may be an argument that aspects of the teaching approach at the time did not accurately reflect the real clinical practice for these conditions. The students also seem to regularly have difficulty with questions regarding seizures in childhood despite there being a strong emphasis on seizure disorders and febrile convulsion in the curriculum. The students may not have grasped the subtle but clinically crucial differences between types of seizure presentation. There are multiple examples above where, despite their inclusion in the curriculum, the students appear to have failed to grasp the true clinical relevance of the condition or presentation. Interestingly, for the questions on which the students performed better than the doctors, the questions were quite specific, and the answer can be found directly in the curriculum. This reflects how students learn with limited clinical experience and is a good example of assessment being a key driver of learning.

### Future recommendations

The concept of a high stake’s assessment with a relatively high passing score breaks from tradition. However, we feel that this model could potentially work very well to ensure that all licensed practitioners have a firm grasp of a finite amount of basic knowledge. This could be considered as part of an undergraduate summative assessment or perhaps as part of a formative assessment when applying for postgraduate paediatric training positions (specialist trainee doctor or resident).

Future research in this area is warranted. Future MAC examinations should have a larger number of test items. This will improve the psychometrics of the examination and will increase coverage of the curriculum. Results from these future studies could help with curriculum development by highlighting specific areas of educational need.

## Conclusion

The MAC examination highlights the difference in the level of knowledge expected of a junior doctor starting work in paediatrics between faculty and non-faculty clinicians and can identify gaps between the current curriculum and the ‘hidden curriculum’ required for real world clinical practice.

The concept of a MAC examination to test basic medical knowledge is feasible. While it will require refinement, whether used as a formative or summative assessment, this is an encouraging first step towards this method of examination.

## Supplementary information

**Additional file 1:** Appendix 1: Item Discrimination Index (D) and Item Difficulty (F) for each MAC test item in each of the study groups.

**Additional file 2:** Appendix 2: Analysis of difficult questions.

**Additional file 3:** Appendix 3: MAC examination.

## Data Availability

The datasets during and/or analysed during the current study available from the corresponding author on reasonable request.

## Abbreviations

SHO: Senior House Officer
MAC: Minimum Accepted Competency
RCSI: Royal College of Surgeons in Ireland
QUB: Queen’s University Belfast
BST: Basic Specialist Training
D: Discrimination Index
F: Facility Index
MCQ: Multiple Choice Question
RCPCH: Royal College of Paediatrics and Child Health
