# Supplementary material for: Minimum accepted competency examination: test item analysis
Source: BMC Med Educ. 2022 May 25;22:400. doi: 10.1186/s12909-022-03475-8 (PMC9131523; doi:10.1186/s12909-022-03475-8)
Supplement: Supplementary file 1 — Additional file 1: Appendix 1: Item Discrimination Index (D) and Item Difficulty (F) for each MAC test item in each of the study groups. [file 12909_2022_3475_MOESM1_ESM.docx]

Appendix 1

Item Discrimination Index (D) and Item Difficulty (F) for each MAC test item in each of the study groups

| **Item number** | **DI RCSI Year 1** | **DD RCSI Year 1** | **DI RCSI Year 2** | **DD RCSI Year 2** | **DD BST** | **DI BST** |
| --- | --- | --- | --- | --- | --- | --- |
| 1 | 0.35 | 0.29 | 0.13 | 0.40 | 0.43 | 0.67 |
| 2 | 0.29 | 0.37 | 0.18 | 0.43 | 0.29 | 0.72 |
| 3 | 0.19 | 0.03 | 0.03 | 0.05 | 0.31 | 0.09 |
| 4 | 0.26 | 0.69 | 0.13 | 0.57 | 0.06 | 0.91 |
| 5 | 0.21 | 0.79 | 0.29 | 0.67 | 0.20 | 0.98 |
| 6 | 0.16 | 0.32 | 0.16 | 0.30 | 0.11 | 0.34 |
| 7 | 0.13 | 0.09 | 0.23 | 0.12 | 0.35 | 0.33 |
| 8 | 0.26 | 0.34 | 0.26 | 0.22 | 0.02 | 0.72 |
| 9 | 0.31 | 0.41 | 0.29 | 0.35 | 0.14 | 0.86 |
| 10 | 0.23 | 0.32 | 0.23 | 0.20 | 0.42 | 0.59 |
| 11 | 0.17 | 0.37 | 0.23 | 0.37 | 0.39 | 0.55 |
| 12 | 0.19 | 0.01 | 0.15 | 0.04 | 0.41 | 0.16 |
| 13 | 0.11 | 0.37 | 0.15 | 0.42 | 0.34 | 0.45 |
| 14 | 0.12 | 0.35 | 0.38 | 0.35 | 0.26 | 0.38 |
| 15 | 0.14 | 0.29 | 0.22 | 0.29 | 0.49 | 0.52 |
| 16 | 0.17 | 0.91 | 0.09 | 0.96 | 0.00 | 1.00 |
| 17 | 0.27 | 0.31 | 0.22 | 0.39 | 0.34 | 0.76 |
| 18 | 0.32 | 0.36 | 0.17 | 0.32 | 0.35 | 0.81 |
| 19 | 0.59 | 0.69 | 0.11 | 0.74 | 0.44 | 0.69 |
| 20 | 0.29 | 0.54 | 0.20 | 0.40 | 0.24 | 0.52 |
| 21 | 0.38 | 0.62 | 0.23 | 0.58 | 0.29 | 0.81 |
| 22 | 0.27 | 0.60 | 0.22 | 0.46 | 0.36 | 0.64 |
| 23 | 0.26 | 0.50 | 0.30 | 0.54 | 0.21 | 0.60 |
| 24 | 0.22 | 0.75 | 0.41 | 0.60 | 0.29 | 0.84 |
| 25 | 0.14 | 0.43 | 0.11 | 0.67 | 0.16 | 0.34 |
| 26 | 0.48 | 0.50 | 0.20 | 0.69 | 0.26 | 0.79 |
| 27 | 0.19 | 0.65 | 0.27 | 0.77 | 0.10 | 0.93 |
| 28 | 0.26 | 0.58 | 0.24 | 0.26 | 0.37 | 0.62 |
| 29 | 0.21 | 0.53 | 0.38 | 0.37 | 0.21 | 0.74 |
| 30 | 0.18 | 0.79 | 0.12 | 0.86 | 0.45 | 0.88 |
