# Supplementary material for: Minimum accepted competency examination: test item analysis
Source: BMC Med Educ. 2022 May 25;22:400. doi: 10.1186/s12909-022-03475-8 (PMC9131523; doi:10.1186/s12909-022-03475-8)
Supplement: Supplementary file 2 — Additional file 2: Appendix 2: Analysis of difficult questions. [file 12909_2022_3475_MOESM2_ESM.docx]

Appendix 2

*Analysis of difficult questions*

Item no.1: candidates failed to identify the significance of the focal aspect of the seizure and therefore the possibility of encephalitis.

Item no.3: candidates unlikely to be familiar with persistent bacterial bronchitis and therefore will not know the appropriate management.

Item no.6: this was an example of a typical viral induced wheeze in practice but perhaps not ‘textbook'.

Item no.7: some candidates did not appreciate the clinical significance of the history whilst others, who may have identified that the child has signs of obstructive sleep apnoea, were unaware of the next most appropriate referral.

Item no.8: this was a straightforward calculation of fluid requirements. Although clearly in the curriculum it is unlikely that the student would ever have actually physically prescribed fluids and therefore may have found this question esoteric in nature.

Item no.10; the premise that the child has a typical febrile seizure and does not require any further investigation should be known by the students

Item no.12: candidates not equipped to identify the clear signs of viral illness (i.e. constellation of symptoms whilst being systemically well) rather than focal symptoms in bacterial infections.

Item no.15: students did not grasp the concept or clinical relevance of corrected gestational age. This may be a good example of ‘knows' versus ‘knows how' level of knowledge.

Item no.18: this question was regarding bronchiolitis. There may be discrepancies between what is taught in lectures, what is read in the textbook and what is seen in clinical practice.

Item no.28: this question was regarding congestive heart failure in children. It is likely that the students have become pre-occupied with considering cyanosis and femoral pulse when considering congenital heart disease and forgetting about the basics.

*The following test items are those in which the students performed better than the doctors:*

Item no.19: doctors may have been thinking that sepsis is more likely than coarctation.

Item no20: this question required pure factual recall of when Cystic Fibrosis screening was introduced in Ireland. This is specifically mentioned in the curriculum for RCSI curriculum.

Item no25: the doctors may have been put off by the term ‘punctum’ but cellulitis is also specifically mentioned in the RCSI curriculum
