# Supplementary material for: Minimum accepted competency examination: test item analysis
Source: BMC Med Educ. 2022 May 25;22:400. doi: 10.1186/s12909-022-03475-8 (PMC9131523; doi:10.1186/s12909-022-03475-8)
Supplement: Supplementary file 3 — Additional file 3: Appendix 3: MAC examination. [file 12909_2022_3475_MOESM3_ESM.docx]

Appendix 3

### MAC examination

Correct answers are highlighted in **bold**

**student no:___________________________**

**Graduate entry student (please circle): Yes No**

**Instructions: There are 30 ‘best of five’ multiple-choice questions. There is one correct answer per question. Please answer all questions. Indicate your answer by circling either a, b, c, d or e.**

1. A three-week-old baby is brought to the emergency department following a seizure. The mother reports that the child went unresponsive and only had shaking of the left arm. This lasted for 15 minutes. Ten minutes later the child had a generalised tonic clonic seizure, and this lasted for 20 minutes eventually stopping when the child received anticonvulsants in the ambulance. What treatment would you give?

a.   Amoxicillin, gentamicin and acyclovir

b. **Amoxicillin, cefotaxime, gentamicin and acyclovir**

c. Cefotaxime and acyclovir

d. Penicillin, amoxicillin and gentamicin

e. Penicillin and gentamicin

2. Which of the following conditions is most commonly associated with an omphalocoele?

1. **Beckwith-Wiedemann syndrome**
2. Trisomy 21
3. Mowat-Wilson Syndrome
4. Turner syndrome
5. VACTERL

3. A six year old boy attends paediatric clinic with a history of persistent chesty cough every day for the last eight weeks. He had a “heavy cold” seven weeks ago. He is slightly off form but otherwise well. His chest x-ray is normal. His respiratory examination is normal. Please choose the most appropriate management plan.

- 1. Admit for observation and request specialist respiratory opinion
  2. Commence a trial of inhaled corticosteroids
  3. **Prescribe a six week course of oral co-amoxiclav**
  4. Reassure that this is a post viral cough which is normal and does not require any further treatment
  5. Take a perinasal swab for pertussis and prescribe a five day course of oral azithromycin

4. A six month old infant attends the paediatric out patient clinic for review. She requires some support to sit and is not able to crawl. She displays a palmar type of grasp. She does not have a hand preference. She is babbling. How would you describe her development?

1. Delayed vision and fine motor
2. Delayed gross motor
3. Delayed speech and language
4. Global developmental delay
5. **Normal development**

5. Which of the following statements is true concerning febrile seizures.

1. Febrile seizures are rare in paediatric practice
2. Intravenous Acyclovir should be prescribed.
3. Lumbar puncture and Cerebrospinal fluid (CSF) analysis should be prioritised.
4. **The majority of seizures resolve without any treatment**
5. They are associated with an underlying brain anomaly

6. An 18 month old boy attends paediatric clinic with a history of five separate episodes of wheeze and cough at night, whilst suffering with coryzal symptoms. The wheeze responded to salbutamol inhaler and a short course of oral steroids prescribed by his GP. He is well between episodes. Please choose the most appropriate management plan.

1. Commence a long acting beta agonist.
2. Continue to treat as before with inhaled salbutamol and oral steroids symptomatically.
3. **Give a 6 month trial of inhaled corticosteroids.**
4. No investigation or treatment is required.
5. Prescribe montelukast (singulair – a leukotriene antagonist)

7. A three year old boy presents to paediatric clinic with a history of snoring at night. Mum is worried that he will “stop breathing in his sleep”. He has a normal respiratory examination. He sleeps 11 hours at night and has a one hour nap during the day. The snoring has been happening for a year now. He has no breathing problems during the day. He does not have any relevant medical history, specifically he does not suffer from tonsilitis. Choose the most appropriate management step.

1. Order a lateral neck x-ray
2. Prescribe nasal decongestants
3. Reassure mum that snoring is very common in boys of this age and that he will grow out of it
4. **Refer him to a respiratory specialist for further evaluation**
5. Refer to ENT for airway evaluation.

8. The approximate maintenance fluid requirement for a 10kg infant expressed as mls/hour is:

1. 30
2. **40**
3. 80
4. 90
5. 100

9. In the management of diabetic ketoacidosis (DKA), which of the following is the most appropriate initial treatment?

1. Intravenous dextrose
2. Intravenous insulin
3. **Intravenous isotonic fluid**
4. Oral potassium
5. Subcutaneous insulin

10. You are meeting with the parents of an 18 month old boy who the previous night had a first generalised seizure following a fever of 39.4 degrees Celsius. He has evidence of otitis media on examination. He is developmentally normal. There is no family history of epilepsy. His neurological examination is normal. The most appropriate advice to parents is:

1. Antipyretics are effective in preventing febrile seizures
2. CT brain is preferable to MRI brain in evaluation of this condition
3. **Electroencephalogram (EEG) is not indicated**
4. Risk of another seizure is approximately 50%
5. The child has a 5% chance of developing epilepsy

11. You have completed the Neonatal Resuscitation programme. Which of the following statements is **least** important in resuscitating a term infant requiring resuscitation?

1. **It is not possible to identify conditions associated with high mortality and poor outcome in which withholding resuscitative efforts may be considered reasonable.**
2. The chest should be permitted to re-expand fully during relaxation, but the rescuer's thumbs should not leave the chest.
3. The primary measure of adequate initial ventilation is prompt improvement in heart rate.
4. To appropriately compare oxygen saturations to similar published data, the probe should be attached to the wrist or medial surface of the palm.
5. Volume expansion should be considered when blood loss is known or suspected and the baby's heart rate has not responded adequately to other resuscitative measure.

12. A 2 ½ year old boy attends the emergency department presenting with a three day history of worsening cough, coryzal symptoms and fever. His temperature is 38.5^o^C in the department, on examination he has wheeze bilaterally and scattered crackles. His O_2_ sats are 96% on room air and he is playing with his toys. His white cell count is 19 (normal range 6-17) and CRP is 26. The chest x-ray reports bilateral patchy consolidation predominantly at the bases. Please choose the most appropriate management plan.

1. Admit to the hospital and commence intravenous antibiotics
2. Admit to the hospital and commence oral antibiotics
3. Arrange a mantoux test
4. Prescribe oral antibiotics and discharge home
5. **Reassure that nil further treatment is required and discharge home**

13. Put the following treatments in order of administration in a child with an anaphylactic reaction (given that the ABC`s are in place):

1. Chlorphenamine, Hydrocortisone, IM Adrenaline, IV fluid bolus
2. Hydrocortisone, IM Adrenaline, Chlorphenamine, IV fluid bolus
3. IM Adrenaline, Hydrocortisone, IV fluid bolus, Chlorphenamine
4. **IM Adrenaline, IV fluid bolus, Chlorphenamine, Hydrocortisone**
5. IV fluid bolus, IM adrenaline, Hydrocortisone, Chlorphenamine

14. An 18 month old child is brought into the resus area of the emergency department. Which of the following would be the best estimated weight for this child?

1. 6kg
2. 8kg
3. **11kg**
4. 13kg
5. 15kg

15. A baby was born at 34 weeks’ gestation. He spent three weeks in the neonatal intensive care unit (NICU) before discharge home. He attends the outpatient clinic for his six week check. What chronological age is this baby at the six week check?

1. 6 weeks
2. 8 weeks
3. 10 weeks
4. **12 weeks**
5. 14 weeks

16. A six month old infant is brought into the emergency department with a fever of 39.2^o^C. The best place to check this child’s capillary refill time is.

1. Forehead
2. **Sternum**
3. Tip of either thumb
4. Tip of left index finger
5. Tip of right index finger

17. A 2 year old boy presents with a temperature of 39.1^o^C. His parents are concerned that he has had some unusual limb movements, which have since discontinued. They show you a video of the event which shows that the boy has jerking of his left arm and appears to have a feared look on his face. Of note, the parents report that he has had recent “flu”. No relevant medical history. Choose the most appropriate advice.

1. Administer buccal midazolam
2. **Admit for further evaluation of this episode**
3. Discharge home and arrange neurological review and electroencephalogram (EEG) as an outpatient
4. Reassure that this is a simple febrile seizure and discharge home
5. Reassure that this is a simple febrile seizure and give parents advice regarding how to manage potential future events

18. Which of the following best describes the initial hospital management of bronchiolitis?

1. Minimal handling, supplemental oxygen as required and amoxicillin
2. Minimal handling, supplemental oxygen as required and salbutamol as required
3. **Minimal handling and supplemental oxygen as required**
4. Nasal continuous positive airway pressure (CPAP) and supplemental oxygen as required
5. Nasal CPAP and nasogastric feeds

19. A 10 day old term infant who has taken their last two feeds poorly attends the emergency department collapsed. The femoral pulses are difficult to palpate. What is the most likely diagnosis?

1. Congenital adrenal hyperplasia
2. **Congenital heart disease**
3. Dehydration
4. Group B streptococcal sepsis
5. Metabolic disease

20. When was newborn screening for Cystic Fibrosis introduced in the Republic of Ireland.

1. July 1995
2. July 2001
3. July 2008
4. **July 2011**
5. July 2013

21. A 7 year old child presents with a non-blanching rash on his lower limbs. This was preceded by a fever and cough for three days. Full blood count shows (normal ranges shown in brackets); haemoglobin 120 g/L (115-155), white cell count 12 (4.5-14.5), platelet count 30 (150-400).  Which of the following is the most likely diagnosis?

1. Acute lymphoblastic leukaemia
2. Cough induced petechiae
3. Henoch schonlein purpura
4. **Idiopathic thrombocytopenic purpura (ITP)**
5. Meningococcal disease

22. Which of the following are **not** cyanotic congenital heart lesions.

1. **Co-arctation of aorta**
2. Ebstein’s anomaly
3. Pulmonary atresia
4. Total anomalous venous drainage
5. Transposition of great arteries

23.      A five-month-old child presents with a sore swollen knee and a temperature of 39.5C. The child still has some varicella lesions, which are not yet crusted over. She has no allergies. She is feeding poorly. What antimicrobial management would you consider in this setting?

1. Intravenous Benzyl penicillin
2. **Intravenous cefotaxime and clindamycin**
3. Intravenous clindamycin
4. Oral cephalexin

e. Oral clindamycin

24. An 18 month old girl presents to the outpatient clinic following her first laboratory proven E.Coli urinary tract infection that was treated with oral co-amoxiclav. Her creatinine was normal. There was a family history of renal dysplasia complicated by renal failure. The most important next step in her management is:

1. Antibiotic prophylaxis
2. Micturating cystourethrogram (MCUG)
3. Refer to a paediatric urologist
4. **Order a renal ultrasound**
5. Repeat urinalysis

25. An otherwise well four year old boy presents with fever and a tender, warm erythematous area with a small punctum above his right knee. Select the condition most characteristic of these findings.

1. **Cellulitis**
2. Cutaneous abscess
3. Ecthyma
4. Impetigo
5. Necrotising fasciitis

26. You are the paediatric SHO reviewing a child with pyrexia of unknown origin in the emergency department. The urinalysis was negative, chest x-ray appears normal, CRP 5, full blood count all within normal limits. The parents insist on going home. Your next line of management should be?

1. Ask the parents to sign a ‘leave against medical advice’ form
2. Commence empiric oral antibiotics and discharge home
3. Discharge and arrange follow up review in one week’s time
4. **Inform your supervising registrar**

e. Request an abdominal ultrasound

27. A two year old girl is brought to the emergency department with a generalised seizure. The nurse informs you that her blood sugar is 1.1 on arrival. Which of the following treatment options is the priority?

1. Diazepam per rectum
2. Intubate the child with rapid sequence induction
3. **Intravenous bolus of 10% glucose**
4. Intravenous lorazepam
5. Phenytoin infusion

28. Which of the following signs are typically associated with congested heart failure.

1. Absent femoral pulses
2. Central cyanosis
3. Peripheral cyanosis
4. **Poor weight gain**
5. Seizures

29. A six month old baby, four days following open heart surgery, is lifeless and has no palpable pulse. A chest lead rhythm trace shows wide complex QRS complexes, absent p waves at a rate of 220 bpm. Which is the best treatment from the options below:

1. External cardiac pacing
2. Intravenous amiodarone
3. Intravenous isoprenaline
4. **Unsynchronised dc shock at 4j/kg**
5. Valsalva manoeuvre

30. What is the average birth weight of a newborn term infant?

1. 0.5kg
2. 1.5kg
3. 2.5kg
4. **3.5kg**
5. 5.0kg
